# Driving health transformation: big pharma’s innovation labs revolution

**DOI:** 10.1186/s12961-025-01415-8

**Published:** 2025-10-23

**Authors:** Galo Peralta, Blanca Sánchez

**Affiliations:** 1 Instituto de Investigación Marqués de Valdecilla (IDIVAL), Santander, Spain; 2 Clinical Pharmacology Service, Hospital Universitario Marqués de Valdecilla (IDIVAL), Santander, Spain

**Keywords:** Innovation labs, Healthcare innovation, Healthcare transformation, Innovation ecosystems, Hospital-based innovation, Experimental spaces

## Abstract

**Background:**

Large pharmaceutical companies are evolving their innovation strategies, moving from closed R&D models towards open, collaborative ecosystems. Innovation labs have emerged as key organizational infrastructures in this shift, designed to accelerate the development, validation and adoption of new healthcare solutions. However, a systematic understanding of industry-led innovation labs remains limited.

**Objective:**

This study aims to comprehensively characterize the structure, strategic focus, activities and outputs of innovation labs promoted by major pharmaceutical companies, and to identify patterns and divergences across different organizational models.

**Methods:**

We conducted a structured literature review in PubMed and an original mapping of innovation labs established by the top 20 global pharmaceutical companies. Data were collected from peer-reviewed publications, official corporate reports and grey literature. Innovation labs were analyzed according to their digital orientation, geographical structure (unicentric versus multicentric), activity domains, stakeholder engagement and innovation outputs.

**Results:**

A total of 102 innovation centres promoted by 14 pharmaceutical companies were included. Most centres demonstrated a strong digital focus, particularly on digital health solutions and remote patient monitoring. Collaboration activities were widely reported (98%), mainly involving universities (92%) and other industries (65%). Support for entrepreneurship was a major theme, reflected in mentoring programs (87%), co-development opportunities (85%) and access to funding (40%). A comparative analysis revealed that multicentric initiatives were significantly more engaged in external collaborations, entrepreneurship promotion and educational activities, and produced higher rates of patents and spin-offs compared with unicentric initiatives. However, unicentric labs were more associated with internal capacity building and early-stage clinical research.

**Conclusions:**

Pharmaceutical innovation labs are pivotal in healthcare transformation, integrating scientific, technological and entrepreneurial approaches. Multicentric and unicentric models offer complementary strengths: multicentric hubs enhance external engagement and scalability, while unicentric labs foster organizational learning and focused research. Understanding and strategically balancing both models could maximize the impact of pharmaceutical innovation infrastructures. Future research should explore longitudinal impacts, patient involvement and the interaction of innovation labs with venture capital ecosystems and regulatory frameworks.

## Introduction

Large pharmaceutical companies are central in advancing medical innovation, primarily through sustained investment in research and development (R&D). In 2022–2023 alone, the world’s top 20 pharmaceutical firms invested a combined total of $145 billion in R&D [1]. This substantial financial commitment underscores the sector’s ongoing efforts to discover new therapies, improve existing treatments and maintain a competitive edge. As a result of these investments, the projected internal rate of return on late-stage R&D portfolios rose to 4.1% in 2023, suggesting renewed momentum in innovation-driven performance [2].

In response to this highly competitive and increasingly complex scientific landscape, pharmaceutical companies are evolving their traditional innovation models, shifting from closed systems towards more open and collaborative approaches based on the principles of open innovation [3–5]. Within this context, innovation labs have emerged as a strategic key organizational infrastructure to accelerate the generation, validation and adoption of new healthcare solutions [6–8].

Building on this foundation, innovation labs act as catalysts for interdisciplinary collaboration and incubators for novel ideas. They provide critical assets – such as specialized facilities, digital infrastructures and expert networks – that accelerate the translation of scientific breakthroughs into practical healthcare solutions [8–12]. By strategically leveraging industry partnerships, these labs connect diverse stakeholders across sectors, fostering synergistic ecosystems that drive efficient healthcare innovation and facilitate regulatory modernization. The integration of advanced digital tools and information technologies within these ecosystems further transforms healthcare delivery, boosting both the efficiency of clinical processes and the quality of patient outcomes [6, 13–19].

The intersection of business acumen and medical expertise within these environments fosters the development of transformative solutions that can significantly improve patient outcomes. Nevertheless, despite their expanding influence, these innovation ecosystems face critical challenges – including ethical complexities, regulatory barriers, sustainability concerns and difficulties in scaling innovations – that may hinder their long-term effectiveness and systemic impact [11]. Understanding these emerging challenges is critical to fully realizing the potential of industry-led innovation infrastructures.

In addition to mapping the organizational structures of pharmaceutical industry-led innovation labs, our study also investigates their orientation towards digital transformation. Digital innovation has become a central driver in pharmaceutical R&D and healthcare ecosystems, encompassing areas such as artificial intelligence for drug discovery [20], data-driven platforms, digital therapeutics and connected care solutions [21]. Given this centrality, one of our research questions explicitly asks: How and to what extent do pharma innovation labs emphasize digital innovation, and what categories of digital activities can be identified? This framing ensures that the analysis of digital orientation is not incidental but reflects a key dimension of the role these labs play within broader health innovation ecosystems.

This study seeks to address that gap by providing a structured analysis of innovation labs promoted by major pharmaceutical companies. It examines them across three interconnected levels – companies, initiatives and individual centres – and highlights both shared patterns and emerging divergences. Although the literature on pharmaceutical innovation is well-established, most studies focus on macro-level dynamics of R&D, costs and external collaborations [22, 23] or on academic-led drug discovery efforts [24]. However, there is a distinct absence of structured academic analysis on industry-led innovation labs in the pharmaceutical sector – entities that embody firms’ strategic shift towards external collaboration and knowledge integration. Indeed, innovation ecosystems in healthcare are documented, but they usually refer to broad collaboration frameworks or academic/public-led initiatives. What remains underexplored in the academic literature are the specific infrastructures promoted by Big Pharma itself – that is, industry-led innovation labs – which are the focus of this study.

Despite their growing presence, there is still no structured academic analysis of industry-led innovation labs in the pharmaceutical sector. Existing studies typically examine macro-level R&D dynamics, costs or external collaborations, or focus on academic/public-sector labs and broad innovation ecosystems, leaving the specific organisational infrastructures promoted by Big Pharma underexplored. This gap – evident in the scarcity of indexed sources and the need to rely on grey literature – underscores the originality and policy relevance of a systematic characterisation of these labs across structures, activities, stakeholders and outputs. Accordingly, we ask:

(R1) How are these labs structured and what is their strategic and operational digital orientation?

(R2) Which activities, stakeholders and outputs characterize them?

(R3) How do unicentric and multicentric models differ in collaboration, entrepreneurship support and innovation outputs?

## Methodology

This study systematically investigates the landscape of industry-led innovation labs in the pharmaceutical sector. The methodology was structured around two main components: a structured literature review and an original mapping of innovation labs promoted by the top 20 global pharmaceutical companies.

## Systematic search and sources

We conducted a structured literature search following PRISMA-S guidelines to identify peer-reviewed publications on pharmaceutical industry-led innovation labs. To balance sensitivity and specificity, we tested three complementary strategies in PubMed, Web of Science (WoS) and Scopus:

1. Broad OR-only strategy. all descriptors were combined with OR, including terms related to innovation labs, pharmaceutical research and ecosystems (for example “innovation labs” OR “pharma innovation” OR “open innovation in pharma”). This strategy generated very large numbers of records, but most were unrelated to industry-led innovation labs.
2. Restrictive AND-only strategy. all descriptors were combined exclusively with AND. This approach produced no results across PubMed, Scopus and WoS, confirming the absence of indexed articles explicitly covering all descriptors simultaneously.
3. Three-block refined strategy. To align the search with our analytical framework (company, initiative/centre and ecosystem framing), we structured the query into three blocks:
4. Block A (initiative/centre level): “innovation lab*” OR “innovation hub*” OR “living lab*” OR accelerator*
5. Block B (company/pharma level): pharma* OR pharmaceutical* OR “drug discovery” OR biopharma* OR “life science*”
6. Block C (ecosystem framing): “open innovation” OR “innovation ecosystem*”

This refined query was designed to maximize the likelihood of retrieving studies specifically addressing pharmaceutical innovation labs within broader ecosystem perspectives.

In addition to the refined three-block strategy, we also performed a broader exploratory search (“pharma AND healthcare AND innovation AND lab*”) across PubMed, WoS and Scopus.

Together, these searches provided a two-step approach: the refined three-block strategy aligned closely with our analytical framework, while the exploratory search served to confirm whether the observed gap was genuine or an artefact of the string. To address this gap, we complemented the database searches with grey literature sources (corporate reports, press releases, case studies) and developed an original mapping of 102 innovation centres promoted by major pharmaceutical companies.

### Mapping of innovation labs promoted by major pharmaceutical companies

The second component involved a targeted review of innovation labs established by the 20 largest pharmaceutical companies on the basis of industry rankings [25].

To ensure comparability and reduce bias, we systematically conducted Google searches using a predefined set of search strings for each company. Specifically, we combined the company name (for example “Pfizer”, “Roche”, “Novartis”) with standardized keywords: (“innovation lab*” OR “innovation hub*” OR accelerator* OR “innovation centre”). Additional variants combined the company name with terms such as “drug discovery”, “digital R&D”, “AI” and “collaboration”. This protocol was applied identically across all twenty companies.

Centres exclusively dedicated to academic research without industry leadership were excluded. Both searches covered the period from 1 January 2013 to 31 December 2023. Data on innovation labs were collected through a multi-source strategy including official corporate websites, annual and innovation reports, press releases, news articles from credible sources and publicly available case studies.

To systematically assess the digital orientation of pharmaceutical innovation labs, we developed a dual-level classification framework distinguishing between strategic digital focus and operational digital activities.

Digital orientation categories. During data extraction we observed that many industry-led innovation labs emphasized digital transformation as a strategic component. To systematically capture this dimension, we developed four non-exclusive categories of digital orientation. These categories were derived inductively from recurrent themes in the dataset of 102 labs, and then triangulated with existing frameworks in the literature on digital health and pharmaceutical innovation ecosystems [20, 26]: (1) primary digital aim – digital innovation explicitly stated as a core strategic goal of the lab; (2) any digital focus – labs with at least one declared digital component, even if not central to their overall mission; (3) aimed at digital health solutions – labs focusing on the design, development or application of digital tools to enhance healthcare delivery or patient care; and (4) digital monitoring – labs incorporating technologies for tracking, remote surveillance or patient monitoring through connected devices or digital platforms.

This combined inductive–deductive approach ensured that the classification was empirically grounded in the data and at the same time conceptually consistent with prior research on digital ecosystems in healthcare.

In parallel, we categorized operational digital activities based on documented outputs and project descriptions, also using a non-exclusive approach. Four categories were defined: (1) pilot digital health solutions, referring to the testing or small-scale deployment of digital innovations; (2) collect and analyze health data, covering activities involving the gathering and analysis of health-related datasets and the use of analytics platforms; (3) integrate digital tools in trials, which included the application of digital instruments or platforms within clinical trials to optimize efficiency, data quality or patient engagement; and (4) test wearables, capturing initiatives that evaluated or implemented wearable technologies for health monitoring or intervention purposes.

Additionally, for the purpose of this study, innovation labs were classified according to their geographical structure: unicentric labs refer to innovation centres with a single operational location, whereas multicentric labs correspond to broader initiatives encompassing multiple geographic sites under a unified strategic framework. This classification aligns with ecosystem literature emphasizing the role of geographic boundaries in shaping ecosystem types and goals [27]. Each company, initiative and individual centre was independently reviewed and coded across these categories based on publicly available information. The categories were not mutually exclusive, allowing multiple classifications per entity.

Data triangulation. To strengthen the validity of our dataset, we applied data triangulation, integrating multiple sources of evidence for each innovation lab identified. This approach reduces the risk of bias associated with relying on a single source and is widely recognized as a strategy to enhance robustness in organizational and innovation research [28, 29]. For each of the top 20 pharmaceutical companies, we systematically combined information from: (a) official corporate websites and dedicated innovation pages; (b) annual reports, innovation reports and investor presentations; (c) press releases and news coverage from credible outlets; and (d) publicly available case studies and secondary reports.

Whenever possible, we cross-verified information across at least two sources before inclusion in the structured database. In cases where discrepancies were observed (for example inconsistent descriptions of a lab’s scope), priority was given to corporate primary sources and complementary sources were used to clarify details. This multi-source strategy ensured consistency across companies and provided a more comprehensive and reliable mapping of industry-led innovation labs.

All information collected was entered into a structured database and systematically coded. We applied a thematic content analysis approach [30]. Coding was first conducted inductively, capturing recurring themes in lab descriptions (for example digital orientation, type of collaboration, geographic scope). These inductive codes were then compared with and refined against existing conceptual frameworks in innovation ecosystem and digital health literature to ensure conceptual consistency.

In this study, we adopt the perspective of health innovation ecosystems [27]. Within this framing, pharmaceutical innovation labs are analyzed as organizational infrastructures embedded in these ecosystems. While in some sections we use the term “platforms” to denote collaborative spaces, we do not employ it in the technical sense of platform economics. Strategic collaborations and entrepreneurship promotion are considered as specific mechanisms operating within these broader ecosystems.

Descriptive statistics, including frequencies and proportions, were subsequently applied to compare the prevalence of digital focus, operational activities and structural types across the different levels of analysis. We applied chi-square tests of independence to assess whether the distribution of categorical variables differed significantly across groups. This test was selected because it is appropriate for analyzing associations between categorical data (for example unicentric versus multicentric structure, presence of specific digital orientations). Results are reported with corresponding p-values.

## Results

### Literature review

**Fig. 1:**
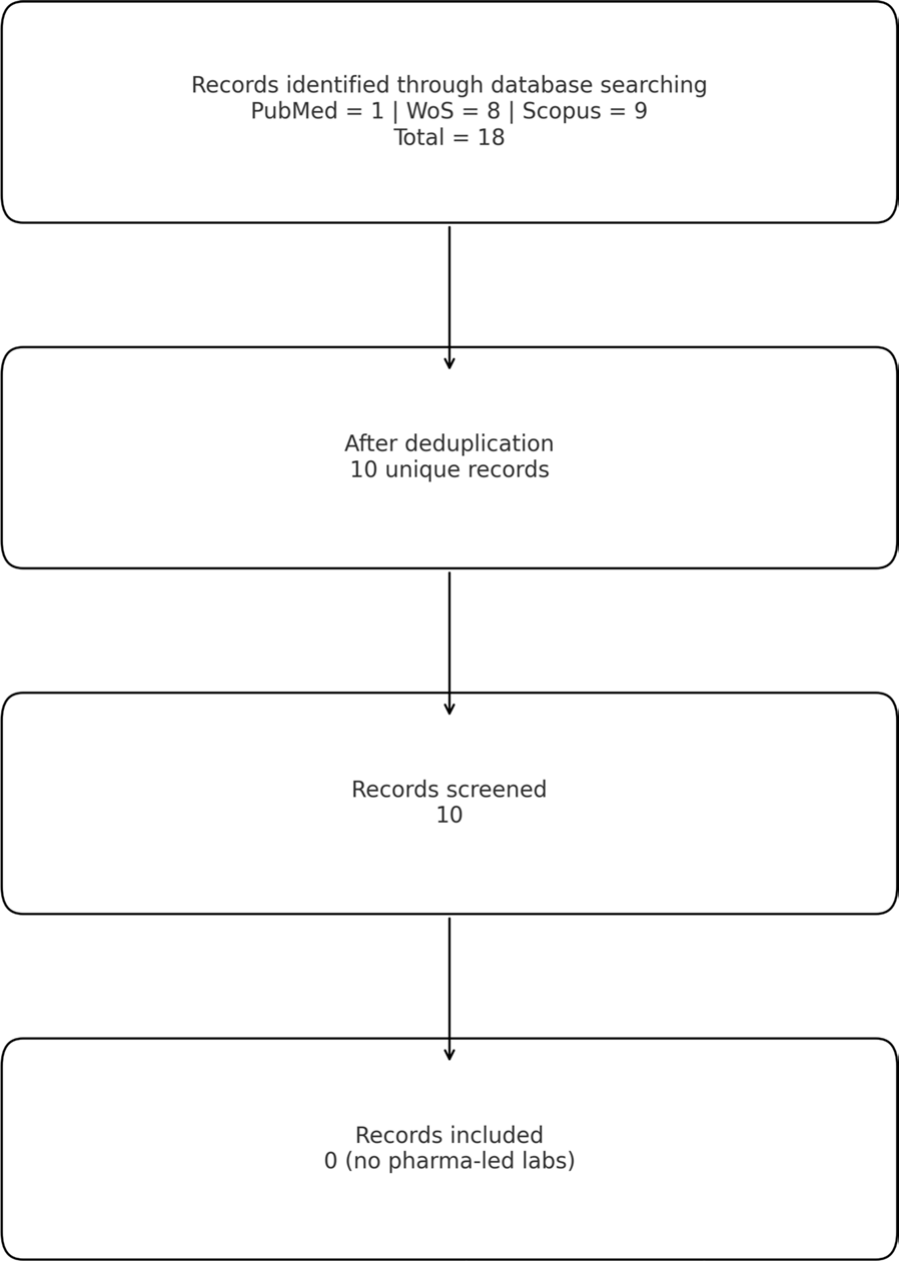
Flow diagram of the literature search and screening process

The systematic search retrieved 1 record in PubMed, 8 in Web of Science (WoS) and 9 in Scopus, resulting in 10 unique publications after deduplication (Fig. 1). Several were theses or grants rather than peer-reviewed articles, and none directly analyzed pharmaceutical industry-led innovation labs.

The few relevant papers addressed broader themes such as open innovation in biopharma, accelerator engagement or ecosystem frameworks, but none provided a systematic, comparative characterization of pharma-led innovation labs (Table 1). This confirmed the absence of indexed academic work in this area and reinforced the novelty of our dataset of 102 centres.

**Table 1:** Publications retrieved through the three-block refined search (PubMed, WoS, Scopus)

Títle	Year	Type of document	Relevance for pharma-led labs
Open innovation hub in AIST [35]	2014	Journal article	Indirect (innovation hub, not pharma-led)
Embracing a Penta helix hub framework for co-creating sustaining and potentially disruptive sterilization innovation…[36]	2025	Review (scoping)	Indirect (framework, not pharma-led)
Implementing open innovation in a closed biopharmaceutical industry [37]	2024	Journal article	Related to pharma, but not labs
A note on corporate open innovation: engagement with startups [38]	2021	Journal article	Indirect (open innovation, no labs)
Introductory overview to special edition – building and leveraging the innovation ecosystem and clusters… [39]	2021	Review	Indirect (ecosystem focus)
A conversation on accelerating innovation in biopharma and life sciences through global collaboration and alliances [40]	2021	Journal article	Indirect (collaboration, not labs)
Open innovation in SMEs: a study of the Swedish bio-pharmaceutical industry [41]	2016	Journal article	Indirect (SMEs, not pharma labs)
IFLEX: a fully open-source, high-density FPGA-based hardware co-processor… [42]	2019	Journal article	Not relevant (technical, not innovation labs)
Clinical pharmacy innovation marathon: Feedback from a successful experience [43]	2025	Journal article	Indirect (clinical pharmacy, not pharma labs)
The innovation ecosystem and implications of the Netherlands [44]	2022	Journal article	Indirect (policy analysis, not pharma labs)

Reference numbers correspond to the main reference list. “Indirect” indicates that the study addressed related concepts (for example ecosystems, open innovation, SMEs or clinical pharmacy) but did not focus on pharmaceutical industry-led innovation labs. “Not relevant” refers to studies outside the scope of innovation labs (for example, technical or hardware-oriented research)

The exploratory search using the broader query (“pharma AND healthcare AND innovation AND lab*”) retrieved 126 records in PubMed, 15 in WoS and 28 in Scopus. After deduplication, 165 unique records remained, with minimal overlap across databases (only four duplicates). However, none of these studies focused on pharmaceutical industry-led innovation labs, as most referred instead to clinical laboratories, hospital-based innovation projects or biomedical research infrastructure.

Taken together, both the refined and exploratory searches consistently highlight the absence of indexed academic studies on pharmaceutical industry-led innovation labs. This scarcity underscores the novelty of our dataset and supports the need for complementary analysis on the basis of grey literature and corporate documentation.

### Analysis of Big Pharma Innovation Labs

Of the 103 innovation centres initially identified within the top 20 pharmaceutical companies, 102 were included in the final analysis, with one excluded due to insufficient available information. The remaining 102 were launched by 14 of the 20 top big pharma companies analyzed (Table 2). In nine cases, the centres were part of a joint initiative under the same pharma, each gathering 2–27 centres and globally 88 of the 102 centres. Overall, 25 different initiatives or centres were identified; 13 (52%) have locations in the USA, 12 in Europe (48%) and 11 (44%) in Asia. Moreover, four (16%) were deployed on three or more continents. The initiatives had a mean age of 5.2 years (range 0–13 years), with 13 initiatives being 5 years old or younger as of 2024.

**Table 2:** Innovation labs of the big pharma and number of locations

Company	Initiative	Locations
AbbVie	AbbVie Innovation Centre	1
AstraZeneca	AstraZeneca Co-Lab	1
AstraZeneca	AstraZeneca’s A.Catalyst Network	27
Bayer	Bayer Co.Labs	2
Boehringer Ingelheim’s	BI X	2
Eli Lilly	Eli Lilly Gateway Labs	4
GSK	GSK AI hub	1
GSK	Stevenage Bioscience Catalyst (SBC):	1
J&J	JLABS	12
J&J	Johnson & Johnson Innovation Centres	4
J&J	Centre for Device Innovation at Texas Medical Centre	1
MSD	MSD IDEA Studios	2
Novartis	Novartis AI Innovation Lab	3
Novartis	Novartis biome	11
Novo Nordisk	Novo Nordisk Innovation Hub & Lab	1
Novo Nordisk	Novo Nordisk Bio Innovation Hub	1
Pfizer	Pfizer Innovation Research (PfIRe) Lab	1
Pfizer	Pfizer Healthcare Hubs	18
Pfizer	Pfizer’s Centre for Digital Innovation	1
Roche	Roche Accelerator	1
Sanofi	Sanofi’s Artificial Intelligence Centre of Excellence	1
Sanofi	Sanofi’s Global Innovation Centre (GIC)	1
Takeda	Centre for External Innovation (CEI)	1
Takeda	Innovation Capability Centres (ICCs)	3
Takeda	Shonan Health Innovation Park (Shonan iPark)	1

To explore these labs’ strategic orientation and activities, we analyzed them across three structural levels – companies, initiatives and individual centres. Table 3 provides a comprehensive overview of the activity domains, strategic aims, collaborative networks, outputs and structural resources across 102 innovation centres, 25 initiatives and 14 pharma companies. The analysis highlights a strong commitment to digital health, entrepreneurship support and external partnerships, with notable variability in patient involvement and internal empowerment efforts.

**Table 3:** Key Activity Domains, Stakeholders and Outputs Across Companies, Initiatives and Centres in Big Pharma Innovation Labs

Activity	Companies (*N* = 14)	Initiatives (*N* = 25)	Centres (*N* = 102)
Domains and fields			
-Digital development	12 (85.7%)	17 (68.0%)	53 (52.0%)
-Devices development	5 (35.7%)	8 (32.0%)	26 (25.5%)
-Drug discovery	8 (57.1%)	10 (40.0%)	59 (57.8%)
Therapeutic focus			
-Specific therapeutic field focus	9 (64.3%)	10 (40.0%)	58 (56.9%)
-Oncology	6 (42.9%)	6 (24.0%)	36 (35.3%)
-Immunology	5 (35.7%)	5 (20.0%)	35 (34.3%)
-Vaccines	3 (21.4%)	4 (16.0%)	4 (3.9%)
-Adherence	4 (28.6%)	4 (16.0%)	31 (30.4%)
Collaboration and stakeholders			
-Collaboration activities	12 (85.7%)	23 (92.0%)	100 (98.0%)
-With universities	12 (85.7%)	20 (80.0%)	94 (92.2%)
-With other industries	11 (78.6%)	18 (72.0%)	66 (64.7%)
-With governments	6 (42.9%)	10 (40.0%)	51 (50.0%)
-With Health professionals	7 (50.0%)	10 (40.0%)	41 (40.2%)
Support for entrepreneurship	13 (92.9%)	18 (72.0%)	91 (89.2%)
-Entrepreneurship support	13 (92.9%)	18 (72.0%)	91 (89.2%)
-Incubation or acceleration	9 (64.3%)	10 (40.0%)	27 (26.5%)
-Funding access	6 (42.9%)	9 (36.0%)	41 (40.2%)
-Mentoring	13 (92.9%)	16 (64.0%)	89 (87.3%)
-Spaces for collaboration	11 (78.6%)	14 (56.0%)	69 (67.6%)
-Market knowledge sharing	10 (71.4%)	11 (44.0%)	86 (84.3%)
-Co-development opportunities	11 (78.6%)	15 (60.0%)	87 (85.3%)
Education and innovation promotion			
-Educational activities	12 (85.7%)	17 (68.0%)	89 (87.3%)
-Universities initiatives	7 (50.0%)	8 (32.0%)	58 (56.9%)
-Networking events	10 (71.4%)	13 (52.0%)	84 (82.4%)
-Seminars	10 (71.4%)	14 (56.0%)	85 (83.3%)
-Mentorship programs	12 (85.7%)	15 (60.0%)	87 (85.3%)
-Work with challenges	8 (57.1%)	11 (44.0%)	65 (63.7%)
-Prizes for innovation	3 (21.4%)	3 (12.0%)	13 (12.7%)
-Online platforms for proposals	9 (64.3%)	12 (48.0%)	82 (80.4%)
Research activities			
-Digital research	7 (50.0%)	14 (56.0%)	18 (17.6%)
-Clinical Research	4 (28.6%)	4 (16.0%)	4 (3.9%)
Resources and infrastructure			
-Multidisciplinary team	12 (85.7%)	19 (76%)	95 (93.1%)
-Offer spaces	11 (78.6%)	15 (60.0%)	68 (66.7%)
-Bio lab spaces	8 (57.1%)	10 (40.0%)	26 (25.5%)
-Prototyping services	4 (28.6%)	5 (20.0%)	16 (15.7%)
Internal/local engagement			
-Internal empowerment activities	4 (28.6%)	5 (20.0%)	7 (6.9%)
-Activities for local integration	10 (71.4%)	16 (64.0%)	87 (85.3%)
Patient involvement			
-Direct patient involvement	2 (14.3%)	3 (12.0%)	13 (12.7%)
-Patient experience focused	8 (57.1%)	12 (48.0%)	82 (80.4%)
Outputs			
-Report significant achievements	9 (64.3%)	9 (36.0%)	50 (49.0%)
-Patents	13 (92.9%)	18 (72.0%)	92 (90.2%)
-Spin offs	13 (92.9%)	20 (80.0%)	97 (95.1%)

Percentages refer to total number of entities per column. Most initiatives include multiple activity areas

A comparative analysis reveals that collaboration is a defining feature of big pharma innovation labs, with over 90% of initiatives and centres reporting partnerships – notably with universities (92%) and other industries (65%). Support for entrepreneurship is another dominant theme, especially in the form of mentoring (87%), collaborative spaces (68%) and access to co-development opportunities (85%).

The primary innovation domain is digital health (in over half the centres), followed by drug discovery and device development. A significant share of labs (57%) focuses on specific therapeutic areas, especially oncology (35%) and immunology (34%).

Patient involvement remained limited (13%), indicating a potential gap in participatory innovation approaches. Education and training activities are present but unevenly distributed, and internal empowerment is rarely cited as a core objective.

**Fig. 2:**
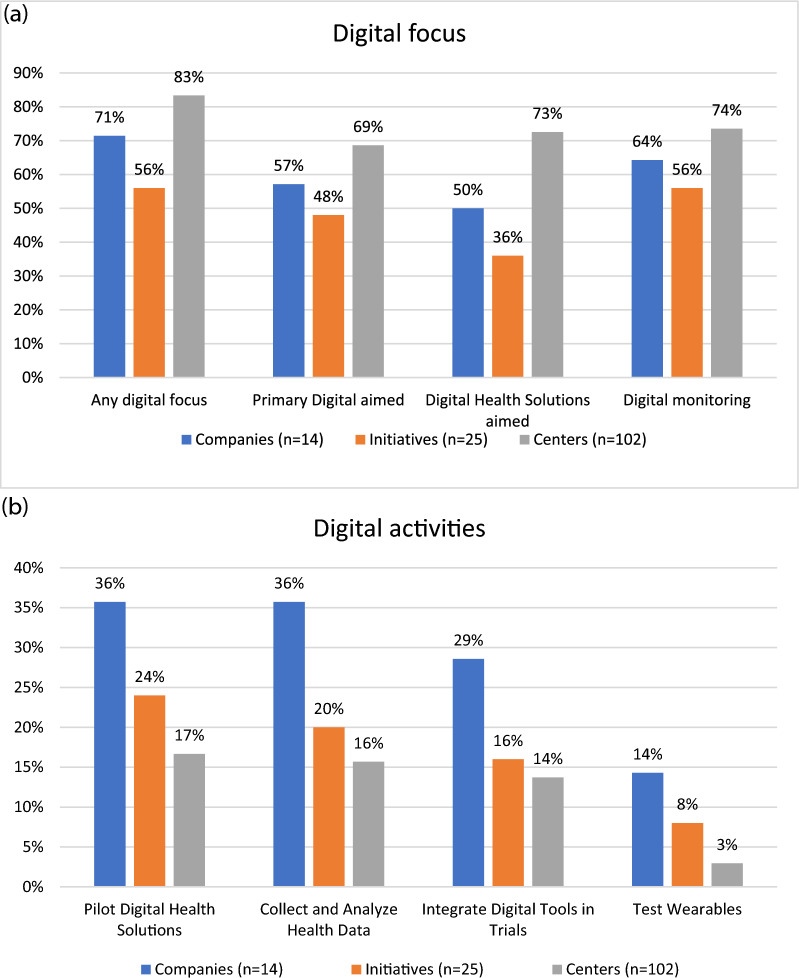
Digital focus (top) and digital activities (bottom) by level of structure

Analyzing digital focus and activities across companies, initiatives and centres reveals notable differences in strategic priorities and implementation levels (Fig. 2). In terms of digital focus, a large proportion of centres (83%) demonstrated some form of digital component, followed by companies (71%) and initiatives (56%). A primary strategic focus on digital innovation was explicitly stated in 57% of companies, compared with 48% of initiatives and 69% of centres. The most common aim was the development of digital health solutions, reported in 73% of centres, 50% of companies and 36% of initiatives. Likewise, digital monitoring activities were reported in 74% of centres, 64% of companies and 56% of initiatives, suggesting a widespread interest in remote surveillance and connected health technologies. Additionally, the mean age of centres with a digital focus (4.35 years) was lower than that of non-digital centres (7.13 years), although the difference was not statistically significant (*t* = − 1.73, *p* = 0.098).

A comparative analysis of unicentric and multicentric initiatives revealed statistically significant differences across several areas. Multi-location centres intensely engaged in external collaboration, entrepreneurship support and educational and innovation promotion activities. In contrast, single-location centres showed more significant involvement in internal capacity building and clinical research (Table 4).

**Table 4:** Activity domains, stakeholder engagement and outputs compared between single-location and multi-location centres in big pharma innovation labs

Activity	Centres with only location (*N* = 14)	Centres with multiple locations (*N* = 88)	*p*
Domains and fields			
-Digital development	10 (71.4%)	43 (48.9%)	0.20
-Devices development	4 (28.6%)	22 (25.0%)	1.00
-Drug discovery	7 (50.0%)	52 (59.1%)	0.73
Therapeutic focus			
-Specific therapeutic field focus	5 (35.7%)	53 (60.2%)	0.15
-Oncology	3 (21.4%)	33 (37.5%)	0.39
-Immunology	2 (14.3%)	33 (37.5%)	0.16
-Vaccines	2 (14.3%)	2 (2.3%)	0.16
-Adherence	2 (14.3%)	29 (33.0%)	0.27
Collaboration and stakeholders			
-Collaboration activities	12 (85.7%)	88 (100.0%)	0.01
-With universities	11 (78.6%)	83 (94.3%)	0.13
-With other industries	10 (71.4%)	56 (63.6%)	0.79
-With governments	6 (42.9%)	45 (51.1%)	0.77
-With health professionals	6 (42.9%)	35 (39.8%)	1.00
Support for entrepreneurship			
-Entrepreneurship support	9 (64.3%)	82 (93.2%)	0.01
-Incubation or acceleration	5 (35.7%)	22 (25.0%)	0.60
-Funding access	5 (35.7%)	36 (40.9%)	0.94
-Mentoring	7 (50.0%)	82 (93.2%)	< 0.001
-Spaces for collaboration	7 (50.0%)	62 (70.5%)	0.23
-Market knowledge sharing	6 (42.9%)	80 (90.9%)	< 0.001
-Co-development opportunities	7 (50.0%)	80 (90.9%)	< 0.001
Education and innovation promotion			
-Educational activities	9 (64.3%)	80 (90.9%)	0.02
-Universities initiatives	4 (28.6%)	54 (61.4%)	0.04
-Networking events	6 (42.9%)	78 (88.6%)	< 0.001
-Seminars	7 (50.0%)	78 (88.6%)	< 0.001
-Mentorship programs	7 (50.0%)	80 (90.9%)	< 0.001
-Work with challenges	5 (35.7%)	60 (68.2%)	0.04
-Prizes for innovation	2 (14.3%)	11 (12.5%)	1.00
-Online platforms for proposals	6 (42.9%)	76 (86.4%)	< 0.001
Research activities			
-Digital research	5 (35.7%)	13 (14.8%)	0.13
-Clinical research	4 (28.6%)	0 (0.0%)	< 0.001
Resources and infrastructure			
-Multidisciplinary team	9 (64.3%)	86 (97.7%)	< 0.001
-Offer spaces	9 (64.3%)	59 (67.0%)	1.00
-Bio lab spaces	6 (42.9%)	20 (22.7%)	0.20
-Prototyping services	4 (28.6%)	12 (13.6%)	0.30
Internal/local engagement			
-Internal empowerment activities	4 (28.6%)	3 (3.4%)	< 0.001
-Activities for local integration	9 (64.3%)	78 (88.6%)	0.05
Patient involvement			
-Direct patient involvement	2 (14.3%)	11 (12.5%)	1.00
-Patient experience focused	5 (35.7%)	77 (87.5%)	< 0.001
Outputs			
-Report significant achievements	4 (28.6%)	46 (52.3%)	0.17
-Patents	8 (57.1%)	84 (95.5%)	< 0.001
-Spin offs	9 (64.3%)	88 (100.0%)	< 0.001

Support for entrepreneurship represented one of the clearest differentiators between both initiatives. Multicentric centres reported more consistent implementation of support mechanisms such as mentoring, co-development opportunities and market knowledge sharing. These structures facilitate not only the incubation of ideas but also their scaling and market readiness. While incubation services and access to funding were observed in both models, structured and network-based entrepreneurship support was markedly more developed in multicentric contexts.

This greater investment in entrepreneurial capacity among multicentric centres was reflected in their innovation-related outputs. They were more likely to produce patents and spin-offs, indicating an enhanced ability to translate innovation efforts into tangible and transferable results. Although both types of centres reported significant achievements, the outcomes associated with multicentric initiatives suggest stronger links to knowledge valorization and external impact.

A comparable trend was noted in the domain of education and innovation promotion. Multicentric centres were more active in organizing educational activities, networking events, seminars and mentorship programs, and promoting university-driven initiatives and innovation challenges. These efforts not only support capacity building within the ecosystem but also create spaces for visibility, collaboration and the circulation of ideas. The use of digital platforms for engaging external proposals further underscores their emphasis on openness and community engagement.

In contrast, unicentric initiatives exhibited a stronger focus on internal capacity building. Activities aimed at empowering internal teams and strengthening organizational competencies were more common in these centres. This may reflect their role as foundational or exploratory units within broader innovation strategies, focusing on early-stage development or cultural transformation.

Finally, clinical research was reported exclusively among unicentric centres. Although the number of cases was limited, this suggests a more direct involvement in context-specific or pilot studies, potentially linked to the proximity and operational flexibility of single-location structures.

These findings reflect two complementary models of innovation labs: multicentric centres, which emphasize ecosystem engagement, entrepreneurship and knowledge transfer and unicentric centres, which are more oriented towards internal development and specialized research. Understanding their distinct contributions can inform more balanced and effective innovation strategies within the pharmaceutical sector.

## Discussion and conclusions

This study offers a structured characterisation of innovation labs promoted by major pharmaceutical companies, combining a scoping review with an original mapping of 102 centres across 14 firms and three analytical levels (company, initiative, centre). To our knowledge, it is the first comparative account that distinguishes systematically between unicentric and multicentric configurations and links structure to activities, stakeholders and outputs. The paucity of indexed work on industry-led labs – evident in our need to triangulate grey literature – underscores both the novelty and the system-level relevance of these findings.

A central finding is the predominance of digital transformation across pharma-led labs, with digital development, digital health solutions and digital monitoring emerging as common pillars. Centres with a digital focus tend to be more recently established, indicating a sectoral pivot towards data-driven R&D, connected care and AI-enabled capabilities; while this pattern reflects a temporal trend rather than a definitive effect size, it aligns with the reconfiguration of innovation portfolios observed in the industry.

In this study, big pharma innovation labs exhibit diverse collaboration activities and stakeholders, reflecting that open innovation and cross-sector collaboration are strategic priorities. This diversity highlights their role as key mechanisms to enhance R&D pipelines, foster partnerships and support pharmaceutical companies in addressing industry challenges with advanced digital capabilities.

This study’s notable contribution is distinguishing between unicentric and multicentric innovation lab models. Our comparative analysis revealed statistically significant differences across several thematic areas, suggesting that structural configuration plays a relevant role in shaping these centres’ innovation approach and performance.

Multicentric centres strongly orient towards external engagement, particularly in collaboration, entrepreneurship support and innovation promotion. These centres were more likely to offer structured mechanisms for mentoring, co-development, market knowledge sharing and open innovation platforms. They also showed greater output in patent generation and spin-off creation, aligning with the broader shift towards open innovation models. Conversely, unicentric centres are more focused environments for experimentation, organizational transformation and early-stage clinical research. Their contained structures allow for deeper integration with corporate culture, rapid iteration cycles and targeted strategic development. Rather than being less developed, unicentric labs fulfil critical functions in nurturing innovation capabilities and fostering internal learning.

Pharmaceutical companies are increasing their R&D investments and reconfiguring how innovation is structured, shifting from closed, internal pipelines to more open, collaborative and decentralized models [5, 45]. Our findings point to the coexistence of two distinct yet complementary innovation strategies within big pharma: one centred on external collaboration and scalability and another more oriented towards internal and organizational development. Recognizing the strategic value of each model may help pharmaceutical companies design more balanced and effective innovation portfolios.

However, despite their promise, innovation labs face substantial challenges. Pharmaceutical R&D continues to experience declining productivity, with the average cost of bringing a new drug to market now exceeding $3.5 billion [46]. Innovation labs, therefore, serve not only as platforms for creative exploration but also as strategic responses to inefficiencies in traditional R&D models [8].

By promoting cross-disciplinary collaboration and breaking down traditional silos, these spaces facilitate the emergence of novel solutions to complex challenges [9, 47]. Prior studies highlight how the combination of structured methodologies with flexibility enhances the innovation potential of diverse teams, enabling organizations to respond more effectively to emerging needs and opportunities [6, 48, 49]. Furthermore, organizations that leverage innovation labs gain a strategic advantage, allowing them to remain competitive and responsive to emerging challenges and opportunities [50]. These insights align with our findings, particularly in multicentric centres, where cross-functional collaboration and knowledge-sharing mechanisms were more consistently reported [15, 51, 52].

Despite their growing impact, industry-promoted health innovation labs encounter significant challenges, particularly in the ideation and development of novel healthcare solutions [15, 53, 54]. These labs often struggle to position themselves effectively within venture capital ecosystems and open innovation platforms, essential for advancing innovative healthcare products towards regulatory approval [5]. Moreover, the complex interplay between marketing strategies and ethical considerations underscores the need for robust governance frameworks that navigate technical, regulatory and ethical landscapes simultaneously [55].

To enhance the impact of innovation labs in the healthcare sector, it is crucial to foster environments that promote strategic collaboration and knowledge sharing among various stakeholders [56–58]. Establishing healthcare hubs can be instrumental in this regard, as they facilitate connections and collaborations among clinicians, researchers, entrepreneurs and industry leaders to address unmet healthcare needs [19, 59, 61]. Beyond their role as platforms for generating new solutions, innovation labs are key mechanisms for knowledge absorption within large pharmaceutical companies. By facilitating structured interactions with external partners and integrating emerging scientific approaches, they enhance the organization’s absorptive capacity – enabling the assimilation, transformation and application of novel knowledge to internal R&D pipelines [62].

From a portfolio management perspective, these insights suggest that pharmaceutical companies have considered a diversified innovation strategy that combines both models. Multicentric hubs can maximize visibility, reach and external input, while unicentric centres can concentrate on depth, continuity and organizational learning. Additionally, investing in clear interfaces and synergies between both types of labs – such as shared knowledge platforms, cross-lab mentoring schemes or aligned governance structures – may enhance overall effectiveness [55].

The findings reveal that patient involvement remains marginal across the observed centres, despite growing attention to user-centred design. Increasing participation from end-users could help ensure that innovation efforts are aligned with real-world needs and expectations, enhancing both impact and adoption [63].

This study has several limitations that should be acknowledged. First, the analysis is based on publicly available information, which may not fully reflect the innovation labs’ internal operations, informal collaborations or unpublished outcomes. As such, some activities may be underreported, particularly those still in early or confidential phases. Second, the study does not include corporate venture capital initiatives promoted by pharmaceutical companies. These investment arms – often operating in parallel with innovation labs – play a significant role in shaping the startup landscape, driving external innovation and expanding the reach of big pharma into adjacent markets. Their exclusion limits the scope of this analysis to structural innovation labs, potentially overlooking important dynamics in the broader innovation ecosystem.

Importantly, reliance on grey literature is both a limitation and a contribution of this study. While it reflects the scarcity of indexed academic sources on industry-led innovation labs, it also enabled us to build an original and systematic dataset that would otherwise remain undocumented. In this sense, grey literature both highlights the academic gap and provides a unique evidence base for policy and systems analysis.

In addition, the cross-sectional nature of the data provides a snapshot rather than a longitudinal view of how innovation labs evolve, merge or dissolve over time. Future research could build on this foundation by incorporating impact evaluations, stakeholder perceptions or longitudinal case studies to assess the sustainability and effectiveness of different lab models. Further work could also explore how innovation labs interact with corporate venture capital initiatives, academic networks and regulatory environments to form integrated innovation strategies in the pharmaceutical sector.

Pharmaceutical innovation labs have emerged as vital mechanisms for accelerating healthcare transformation, blending scientific, technological and entrepreneurial approaches. Recognizing the distinct contributions of unicentric and multicentric models – and fostering strategic alignment between them – will be critical for companies seeking to sustain innovation leadership.

Future research could build on this foundation by incorporating impact evaluations, stakeholder perspectives and longitudinal analyses to assess the sustainability of different lab models over time. Additionally, exploring the interaction between innovation labs, corporate venture capital initiatives, academic ecosystems and regulatory environments could provide a more integrated understanding of pharmaceutical innovation strategies.

From the perspective of dynamic capabilities [64], pharma innovation labs can be interpreted as organizational vehicles for sensing, seizing and transforming. By scouting emerging technologies, startups and digital solutions, they enable sensing of external opportunities; through partnerships, co-creation processes and joint initiatives, they support seizing of collaborative knowledge; and by embedding digital tools, piloting new care models or scaling innovative processes, they contribute to transforming internal corporate capabilities. This framing reinforces their role not only as spaces for experimentation, but also as infrastructures for knowledge transfer and capability building within health innovation ecosystems.

On the basis of the outputs identified in our analysis, several concrete recommendations emerge. First, patient and professional engagement should be strengthened, as it remains uneven across labs, yet represents a critical mechanism for both innovation relevance and adoption. Second, while digital innovation is widespread, a stronger focus on tangible outcomes – including validated services, clinical trial tools and health quality-of-life improvements – would enhance the measurable impact of labs. Third, a more systematic approach to evaluating outputs and disseminating learnings across networks would help overcome the current fragmentation and reinforce the transformative role of pharma innovation labs. Together, these strategies would enable industry-led labs to realize their full potential as dynamic, adaptive infrastructures driving collaborative health innovation.

Our findings underline that pharma-led innovation labs function as systemic actors with direct implications for health policy and governance. They consistently collaborate with universities, industry partners, governments and healthcare professionals, positioning these infrastructures as boundary-spanning interfaces where corporate R&D agendas intersect with public health priorities. Their emphasis on entrepreneurship and education shows that they not only generate patents and spin-offs but also contribute to capacity building across health systems. At the same time, the limited involvement of patients highlights an area where closer alignment with policy objectives – particularly participatory and user-centred innovation – remains critical. Strengthening these connections could accelerate regulatory adaptation, support digital health integration into care pathways and inform national and regional policies aimed at building more resilient, efficient and patient-centred health systems.

## Data Availability

The datasets used and analysed during the current study are available from the corresponding author upon reasonable request.
